# Female intrasexual competitiveness interacts with body mass index to predict willingness to use a risky diet pill

**DOI:** 10.3389/fpsyg.2023.1167115

**Published:** 2023-06-01

**Authors:** Steven Arnocky, Hillary Brennan, Brittany Denomme, Adam C. Davis

**Affiliations:** ^1^Department of Psychology, Nipissing University, North Bay, ON, Canada; ^2^Department of Social Sciences, School of Access, Language, and Preparatory Studies, Canadore College, North Bay, ON, Canada

**Keywords:** intrasexual competition, appearance enhancement effort, weight loss, eating disorders, dieting, diet pill, weight control, female competition

## Abstract

**Introduction:**

Previous research has highlighted the putative role of intrasexual competition (IC) in predicting women’s body dissatisfaction, weight loss effort, and, at its extreme, eating disorders. However, extant research reporting on these links is limited by its exclusion of potential confounds, including psychopathologies such as depression. Moreover, it is presently unclear whether women higher in body mass index (BMI) may be more prone to the influence of IC in taking dieting risks.

**Methods:**

To address these gaps in the literature, 189 young adult women completed measures of IC, depressive symptoms, willingness to use a risky diet pill, and had their height and weight measured.

**Results:**

Results showed that IC interacted with BMI to predict willingness to use a risky diet pill, such that women high in both IC and BMI were most likely to take the risky diet pill. Further exploratory analyses considering potential directional links between BMI and depression supported mediating roles of depression (from BMI) and BMI (from depression) in predicting willingness to use a risky diet pill.

**Discussion:**

Results suggest that links between IC and dieting risks may be moderated by women’s BMI, and that these links hold when considering depressive symptoms. Future longitudinal research would benefit from a better understanding of the potential directional links between BMI, depression, and diet pill use.

## Introduction

Men more than women exhibit a robust mate preference for physically attractive partners (Walter et al., 2020). Among females, physical attractiveness is contributed to by a low waist-to-hip ratio and a low (but healthy) body mass index (BMI; see Arnocky et al., 2014; Davis and Arnocky, 2022, for reviews). Therefore, it is unsurprising that women more than men universally report exerting effort aimed at enhancing their physical appearance, including dieting for improving their physical attractiveness (Kowal et al., 2022). One risky weight control behavior is the use of diet pills. Upwards of 8% of healthy weight adolescent girls and young women report using diet pills, with use being predicted by poor body image and self-perceptions of higher weight (Thorlton et al., 2014). Estimates of diet pill and other diet-aid usage may be even higher among college-aged women, with some studies showing rates as high as 32% and correlations with body weight and shape concerns (Celio et al., 2006). Moreover, in a large study of over 10,000 females, researchers found those who used diet pills were at increased risk for subsequent eating disorder diagnoses within 1–3 years (Levinson et al., 2019). Youth using diet pills and other weight loss supplements are also at an increased risk for hospitalization, disability, and even death (Rao et al., 2017).

It is not surprising, then, that considerable work has been dedicated to understanding the etiology of body image disturbance and weight control behavior, including the use of diet pills. Much research has highlighted the role of proximate factors (i.e., immediate causal mechanisms), including social norms, media influence, family influence, and bullying (e.g., Stice, 2002). In addition to these influences, evolutionary psychologists have considered more ultimate explanations, such as competition among same-sex individuals for mates and mating relevant resources [i.e., intrasexual competition (IC); see Locke and Arnocky, 2020, for review]. Researchers have also highlighted the importance of considering the interactions between sociocultural (e.g., ethnicity) and evolutionary factors influencing body image and appearance enhancement behavior (Frederick and Reynolds, 2022). The evolutionary model with the most empirical support suggests that a drive for thinness serves to outrival other women in the domain of physical attractiveness, as a form of IC (Abed, 1998; Mealey, 2000; reviewed in Davis and Arnocky, 2022). However, extant research linking weight-loss effort to IC is equivocal. First, it is largely limited to the study of extreme symptoms of body image disturbance and weight control, such as disordered eating. Second, these studies lack control of potential confounding variables such as comorbid psychopathology, particularly disordered mood, which can be complicit in both intrasexual competitiveness and body image disturbance. Third, studies have been largely limited to demonstrating links between IC and body image, dieting, and eating pathology, without considering how IC might interact with objective markers of weight-related attractiveness, such as body mass index (BMI). For instance, it is possible that the utility of risky weight control effort might be higher among intrasexually competitive women who are simultaneously higher in BMI, but less so among women with lower BMI. The goal of this research was to address these gaps by examining whether individual differences in IC interact with BMI to predict willingness to use a risky diet pill, controlling for symptoms of depression.

### Intrasexual competitiveness

IC involves rivalry with members of the same sex over access to mates, territory, status, or other reproductively relevant resources (Buss, 1988; Buunk and Fisher, 2009; Arnocky and Vaillancourt, 2017). Often, the focus of competition in one sex revolves around traits or resources that are desired by the opposite sex (Buss, 1988; Arnocky, 2018). Given the importance of partner physical attractiveness to male mate choice (Buss, 1989), it is not surprising that researchers have considered some of the riskiest examples of women’s appearance enhancement from the perspective of IC (Dubbs et al., 2017; Davis and Arnocky, 2022, for review). Buss (1988) identified that women enhance their appearance to make themselves more attractive than same-sex rivals more than men do. Such effort can extend into costly and physically risky behavior, such as cosmetic surgery, particularly when expressing a short-term mating orientation (Bradshaw et al., 2019), and when they are higher in IC (Arnocky and Piché, 2014). Women also use appearance enhancement more than men as means of retaining desired mates (i.e., mate retention; Buss, 1988; Albert and Arnocky, 2016).

### Intrasexual competition and body image disturbance, dieting, and eating pathologies

One realm of female appearance enhancement effort involves body image, weight loss, and disordered eating. Some researchers have argued a drive for thinness allows females to outcompete rivals in displaying a phenotype that is attractive to men (Mealey, 2000; Vaillancourt, 2013). Eating disorders would then be an extreme example of “runaway” female IC, whereby body dissatisfaction, the drive for thinness, and weight control behavior manifests into the maladaptive psychological and behavioral characteristics of contemporary eating disorders (Abed, 1998; Faer et al., 2005; Abed et al., 2012). Indeed, many women in developed nations believe that men prefer a much thinner body size than they report preferring, which contributes to these deleterious outcomes (Fallon and Rozin, 1985; Cohn et al., 1987; Johnson and Engeln, 2021).

When examined directly, studies have linked women’s IC with a host of variables that are relevant to body image and weight control. For example, Faer et al. (2005) found that IC for mates predicted body dissatisfaction (see also Hendrickse et al., 2017), drive for thinness, anorexia, and bulimia. Similarly, Abed et al. (2012) found that IC positively predicted disordered eating behavior. This link has also been supported experimentally. For example, Li et al. (2010) found that IC cues (seeing an image of a high-status and competitive rival) led to more body dissatisfaction and more restricted eating attitudes in heterosexual, but not homosexual, women. Similarly, Boyer et al. (2021) found that women’s IC predicted unhealthy eating attitudes and weight control (including, but not limited to, diet pill use) via indirect effects on social comparison and poor body image. Hill and Durante (2011) showed that women who were induced with either mating motives or IC were more willing to take a diet pill known to confer risk of serious medical side effects. A follow-up study found that these links were mediated by a reduction in the perceived health risks associated with the behavior (Hill and Durante, 2011).

Taken together, this body of research suggests that there is an association between IC and women’s body dissatisfaction, disordered eating, and diet pill use. However, there are some limitations. First, the abovementioned studies have generally failed to account for other variables, such as indicators of body fat, as well as comorbid psychopathologies such as depression, that have been linked either conceptually or empirically to both IC and body dissatisfaction and eating disorders. One notable exception is a study by Ferguson et al. (2011) who exposed women to an attractive female confederate who was dressed in a manner that accentuated her thin, attractive physique versus in baggier clothing, and with or without an attractive male present. This ostensibly primed IC in the participants. Results indicated that body dissatisfaction was more pronounced when women were exposed to a rival wearing clothing that accentuated her thin figure, especially when coupled with an attractive man, over and above the influences of BMI, depression, and anxiety (Ferguson et al., 2011). However, the researchers did not measure whether their manipulation increased IC in their participants. A follow-up correlational study showed no relationship between a measure of enemy status of other women (rating enemies lower on positive characteristics, such as attractiveness) and body dissatisfaction. Another cross-sectional study found that “peer competitiveness” correlated with body dissatisfaction, over and above the influence of BMI and depression (Muñoz and Ferguson, 2012). However, this study measured competitiveness as feelings of inferiority in comparison to other women (e.g., being anxious about one’s relative appearance, feeling weaker or more timid than other females), which narrowly maps onto one aspect of women’s IC (Albert et al., 2022) and does not appropriately capture the extent to which one holds a competitive orientation toward other women (Buunk and Fisher, 2009). Second, it is unclear whether IC broadly predicts women’s unhealthy weight management, or whether that link is contingent upon one’s current level of body fat.

### Body fat, intrasexual competitiveness, and body dissatisfaction

Researchers have previously predicted links between IC and body fat in women. Some researchers have reasoned that low BMI should correlate with greater IC, because “women are eager to emphasize their attractiveness and compete with rivals in displaying these cues” (Fernández et al., 2014, p. 436). Another possibility is that among young adult women who are in their reproductive prime, higher BMI may predict greater IC because those of lower mate value must expend more effort competing for mating opportunities (see Arnocky et al., 2012). Some indirect evidence supports this. For instance, Gallup and Wilson (2009) found that higher BMI women were more aggressive, and most of women’s aggression (85%) was directed against other women. However, in the only study to examine this link directly, Fernández et al. (2014) did not observe a relationship between BMI and IC in their sample of young Chilean women. Whereas potential links between BMI and IC require further investigation, measures of body fat including BMI and skin fold have been robustly linked to women’s body dissatisfaction, eating disorders, and diet pill use (e.g., Boutelle et al., 2002; Reba-Harrelson et al., 2008; Laska et al., 2012; Lucretia, 2017). Another possibility is that these variables would interact with one another in predicting weight loss effort. Because appearance enhancement practices are costly and sometimes risky, women high in IC who are already thin would benefit less from invoking these risks than women who are heavier. Conversely, women who are heavier but who are not high in IC should presumably be less motivated to engage in risky weight loss efforts, relative to those high in BMI and IC.

### Negative mood, intrasexual competitiveness, and risky weight loss

Negative mood is a potential confound that could account for links between IC and body dissatisfaction. First, depression has been found to correlate positively with IC in one study (Mafra et al., 2021). Second, depressed individuals believe themselves to be less attractive and are less satisfied with their bodies compared to non-depressed individuals, even though ratings made by third parties demonstrated no objective differences in attractiveness between these groups (Noles et al., 1985). This can perhaps manifest as body dissatisfaction and weight control behavior via diet pill use. Research from Canada has shown that diet pill use is about four times higher among depressed women compared to the underlying population rate (Patten, 2001). Generally, this link is considered in clinical literature from the perspective that some diet pills could lead to or exacerbate depression. Accordingly, it is plausible that the relationship between IC and body dissatisfaction and unhealthy weight control efforts could be due to negative mood as a common underlying factor. It is also plausible that depression may play a more directional role in diet pill use. This directional role appears to be reciprocal: higher BMI adults are at greater risk of developing depression in their lifetime, but also, depressed individuals are at greater risk for increasing BMI (Luppino et al., 2010). Accordingly, we also tested two exploratory moderated mediation models for the sequential prediction of BMI from depression and vice versa.

### The present study

The present study investigated whether the relationship between women’s IC and BMI interact to predict willingness to use a risky diet pill, and whether this finding holds in light of depressive symptoms. We were particularly interested in willingness to use a risky diet pill given that beyond its association with IC (Hill and Durante, 2011), previous research has also linked diet pill use to depression (e.g., Patten, 2001), eating disorders, and higher BMI (Boutelle et al., 2002; Reba-Harrelson et al., 2008; Laska et al., 2012; c.f. Stephen et al., 2014). For example, Harring et al. (2010) found that higher BMI women were higher in depression and in their use of diet pills. We anticipated that, in line with past research, heavier women would be more willing to use a risky diet pill. However, following Hill and Durante (2011), we further anticipated that IC would also predict a willingness to use a risky diet pill, and that IC and BMI would interact, such that women high in both IC and BMI would be most interested in taking the risky diet pill, and that this link would hold controlling for depressive symptoms.

## Methods

### Participants

As part of a larger survey on female mating and competitive behavior, 189 Canadian participants aged 17–37 were recruited from Nipissing University (*M_age_* = 20, SD = 2.60). This sample allowed for detection of a small effect (*f *^2^ = 0.02; Cohen, 1988) with 80% power and an alpha of 0.05. The ethnic distribution of participants was predominantly Caucasian (90%), with a minority identifying as Asian (2%), South Asian (3%), Southeast Asian (1%), Arab/West Asian (1%), Black (2%), Indigenous (8%), and Latin (2%).^1^ Participants were remunerated with partial course credit via the University’s online research participation system.

### Procedure and measures

Participants had their height, weight, and BMI measured using a Detecto Apex-SH digital clinical scale with a sonar height rod. Then participants were asked to complete the following self-report measures:

#### The intrasexual competition scale

The 12-item Intrasexual Competition Scale (ICS; Buunk and Fisher, 2009) was used as to assess inter-individual variability in the proclivity to compete with same-sex others for mates and mating resources (e.g., “*I can’t stand it when I meet another woman who is more attractive than I am*”). Participants responded to items using a seven-point Likert-type scale ranging from 1 = *not at all applicable* to 7 = *completely applicable*. Items were averaged to create a mean scale score, with higher scores describing higher intrasexual competitiveness, which demonstrated good internal consistency, α = 0.88.

#### Depressive symptoms

Depression was measured using the 13-item adult short version of the Mood and Feelings Questionnaire (MFQ; Costello and Angold, 1988). The MFQ is used as a screener for depression (e.g., “*I felt miserable or unhappy*”) and has demonstrated validity in non-clinical samples (Thabrew et al., 2018). Participants responded to items along a three-point Likert-type scale ranging from 0 = *not true* to 2 = *true*. Scores were summed, with higher scores indicating greater depression. The MFQ demonstrated good internal consistency, α = 0.91.

#### Attitude toward risky diet pill

Participants rated their interest in taking a diet pill known to cause heart problems later in life, using the item: “*How interested would you be in taking a diet pill, which has shown to be effective for weight loss, but which may cause heart problems later in life?*” Participants also completed a control risk item involving willingness to pain in an unventilated room. Endorsement was rated along a seven-point Likert-type scale ranging from 1 = *not at all interested* to 7 = *very interested* (Hill and Durante, 2011).

## Results

Descriptive statistics and bivariate correlations among study variables are presented in Table 1. Both risky diet pill use and body dissatisfaction correlated positively with IC, supporting previously observed links. However, both BMI and depression scores also positively correlated with IC and diet pill use, highlighting the possibility that the links between IC and unhealthy weight control could be an artifact of these variables.

**Table 1:** Descriptive statistics and bivariate correlations between study variables.

	*n*	*M*	*SD*	Min	Max	1	2	3	4
1. Intrasexual competition	186	2.42	0.93	1.00	5.50	—			
2. Depression	187	8.71	6.13	0.00	26.00	0.26^**^	—		
3. Risky diet pill	186	1.66	1.30	1.00	7.00	0.31^**^	0.37^**^	—	
4. Risk control item	188	3.12	2.12	1.00	7.00	0.11	0.06	0.01	—
5. Body mass index (BMI)	186	24.6	5.56	15.5	53.21	0.15^*^	0.28^**^	0.35^**^	−0.01

Bivariate correlations significant at ^*^p < 0.05 and ^**^p < 0.01.

**Figure 1:**
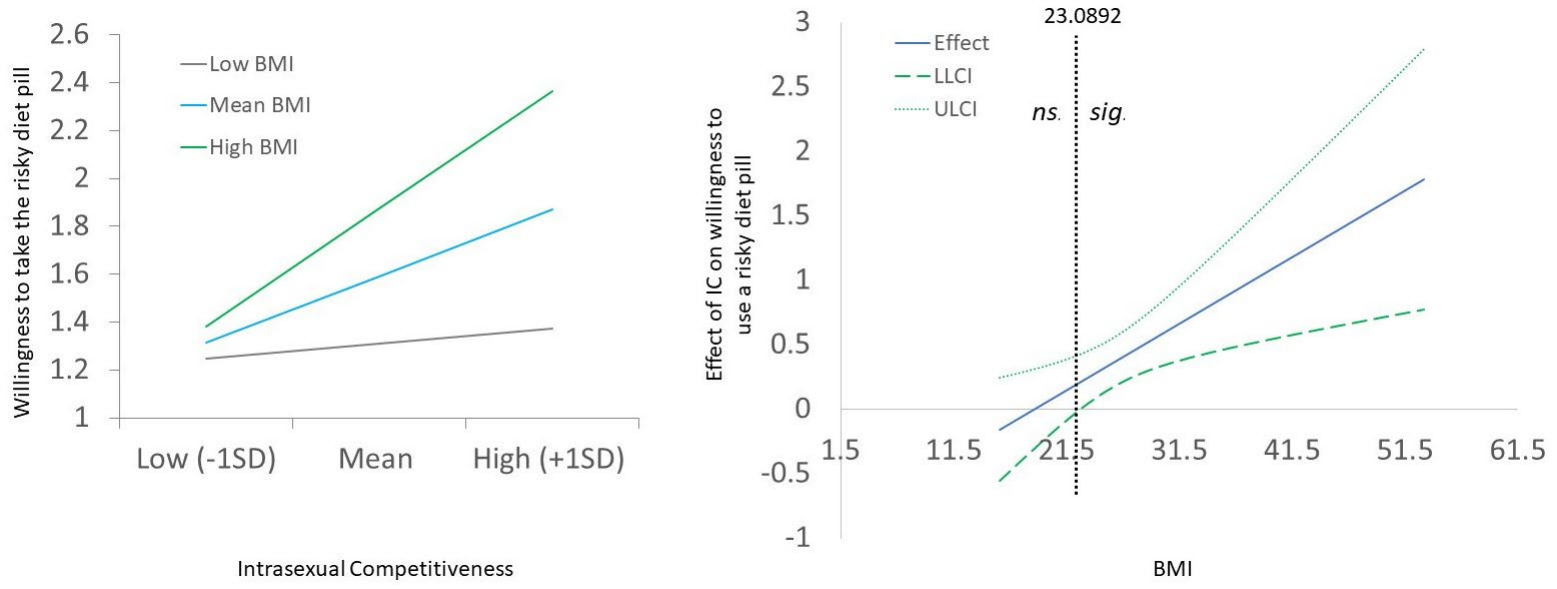
Left panel depicting BMI × Intrasexual competitiveness interaction. Right panel depicting floodlight analysis for determining range of significance for the interaction. ns., the range whereby the interaction effect in statistically non-significant, sig., the point whereby the interaction becomes statistically significant (indicated by the dashed line). LLCI and ULCI, the 95% lowerbound and upperbound confidence intervals, respectively.

To test the simple moderation model, we used Model 1 in the PROCESS macro for SPSS (Hayes, 2013). The macro used ordinary least squares (OLS) regression and bootstrapping (5,000 in this study) to test the moderation model. Depressive symptoms were entered as a covariate, IC was entered as the predictor variable, BMI as the moderator variable, and willingness to use a risky diet pill as the dependent variable. The Johnson-Neyman technique (Aiken and West, 1991) was further used to assess the ranges within which the moderation was significant. Cases with missing values were excluded listwise (final *n* = 179). Model statistics were identical with and without mean-centering the variables; here, we present the findings without mean-centering to aid interpretability of the figures. Results found initial main effects of depressive symptoms (*B* = 0.05, *SE* = 0.02, *t* = 3.52, *p* = 0.0006), IC (*B* = 0.30, *SE* = 0.10, *t* = 3.10, *p* = 0.002), and BMI (*B* = 0.05, *SE* = 0.02, *t* = 3.30, *p* = 0.001) in predicting willingness to use the risky diet pill. The IC × BMI interaction was statistically significant (*B* = 0.05, *SE* = 0.02, *t* = 2.85, *p* = 0.005). Specifically, IC predicted willingness to use the risky diet pill among women scoring either average (*B* = 0.30, *SE* = 0.10, *t* = 3.10, *p* < 0.002) or high (+1 *SD*) in BMI (*B* = 0.59, *SE* = 0.13, *t* = 4.62, *p* < 0.0001), but not for women scoring low (−1 *SD*) on BMI (*B* = 0.02, *SE* = 0.15, *t* = 0.13 *p* < 0.90; Figure 1, left panel). Deconstruction of the interaction showed that the moderation effect was significant for BMI values above the value of 22.74 (Figure 1, right panel), which suggests a significant effect of IC upon willingness to use a risky diet pill when BMI is in the mid-normal range, becoming more significant from the initial overweight range through class 1–3 obesity.

**Figure 2:**
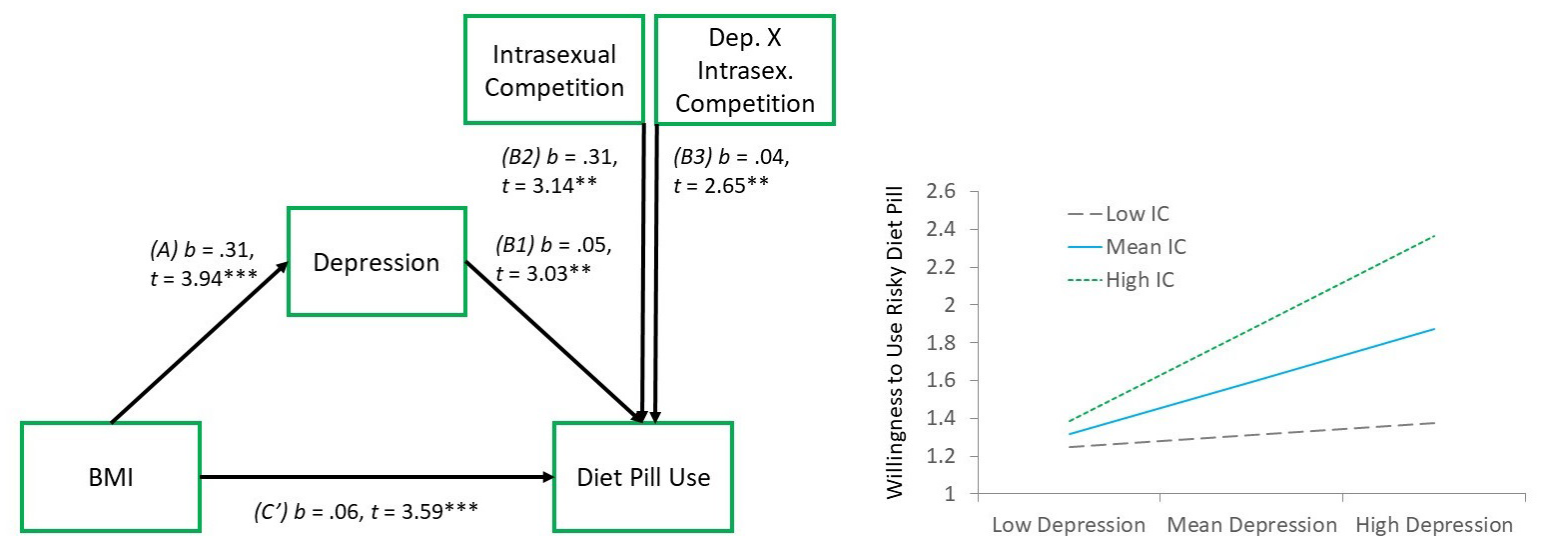
Moderated mediation model 1. Left panel reflecting the model paths, whereby *b*, unstandardized regression coefficient, *t*, standardized *t*-value, ^***^value of *p* < 0.001, ^**^value of *p* < 0.01. Right panel reflecting the depression × intrasexual competition interaction effect.

Next, we tested two exploratory hypotheses based on reviewer recommendations, whereby depression was considered as a directional variable in the model, instead of just as a covariate. In the first moderated mediation model (PROCESS, model 14; Hayes, 2013), we examined whether BMI predicted willingness to use the risky diet pill, with this relationship being mediated by depressive symptoms. The strength of this mediation effect was expected to be moderated by IC. Results showed support for the model via a significant index of moderated mediation, (*B* = 0.01, *SE* = 0.001, LLCI *=* 0.001, ULCI = 0.030). Specifically, the indirect (mediation) effect was not significant at low (−1 *SD*) levels of IC (*B* = 0.003, *SE* = 0.01, LLCI = −0.010, ULCI = 0.023), but was at mean (*B* = 0.014, *SE* = 0.01, LLCI = 0.002, ULCI = 0.034), and high (+1 *SD*; *B* = 0.03, *SE* = 0.01, LLCI = 0.010, ULCI = 0.049) levels (see Figure 2).

**Figure 3:**
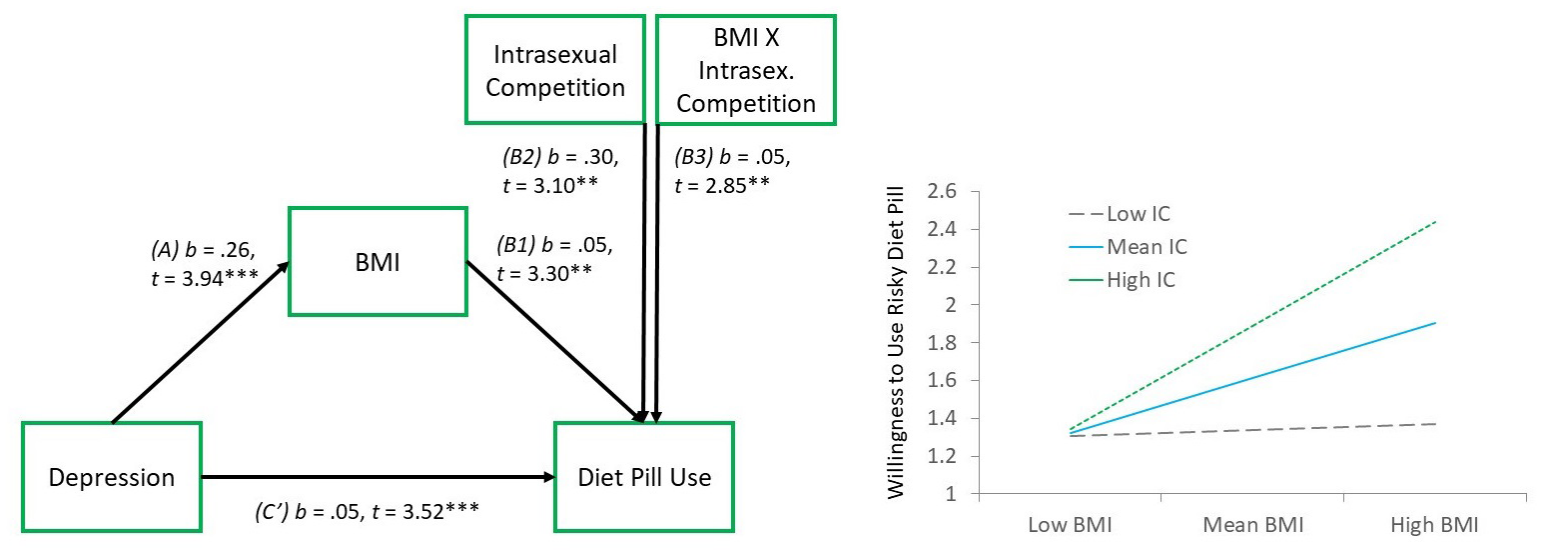
Moderated mediation model 2. Left panel reflecting the model paths, whereby *b*, unstandardized regression coefficient, *t*, standardized *t*-value, ^***^value of *p* < 0.001, ^**^value of *p* < 0.01. Right panel reflecting the BMI × intrasexual competition interaction effect.

We then tested a slightly varied model with depression entered as the predictor variable and BMI as the mediator. Results were nearly identical, with support for the moderated mediation model via a significant index of moderated mediation, (*B* = 0.01, *SE* = 0.006, LLCI *=* 0.003, ULCI = 0.028). Again, the indirect (mediation) effect was not significant at low (−1 *SD*) levels of IC (*B* = 0.002, *SE* = 0.01, LLCI = −0.014, ULCI = 0.014), but was at mean (*B* = 0.014, *SE* = 0.01, LLCI = 0.005, ULCI = 0.025), and high (+1 *SD*; *B* = 0.03, *SE* = 0.01, LLCI = 0.010, ULCI = 0.045) levels (see Figure 3).

## Discussion

Previous research has reported empirical links between women’s intrasexual competitiveness (IC) and measures of body dissatisfaction, eating disorders, and willingness to use a risky diet pill (e.g., Faer et al., 2005; Hill and Durante, 2011; Abed et al., 2012). This suggests that women’s psychology surrounding their weight loss behavior is driven, in part, by the desire to outcompete same-sex rivals for mates and reproductively relevant resources. We extended this line of inquiry by exploring whether IC positively predicted desired willingness to use a risky diet pill while controlling for depressive symptoms, which has previously been linked to both IC and extreme weight control behavior. Moreover, we expected that IC would interact with BMI to predict willingness to use a risky diet pill.

In support of our hypothesis, women who were high in both IC and BMI were the most willing to use a risky diet pill, controlling for depressive symptoms. IC was unrelated to willingness to use the risky diet pill at low BMI (−1*SD*). The Johnson-Neyman floodlight figure demonstrated that the relationship between IC and willingness to use a risky diet pill became statistically significant around a BMI of 23, with the effect becoming highly statistically significant at or above a BMI of 25. This finding suggests that IC may not be uniformly linked to risky weight management behaviors, but rather may be reliant also upon cues to the need to lose weight in order to outcompete same-sex rivals in the domain of physical attractiveness. This finding supports to the notion that IC predicts an approach of altering one’s appearance to satisfy adaptive mating goals. Research supports IC being responsible for a drive for thinness, weight control, and disordered eating behavior as a method of outcompeting rivals (Abed, 1998; Faer et al., 2005; Abed et al., 2012; Locke and Arnocky, 2020). Additionally, previous research has revealed women’s tendency to overestimate males’ desire for thinness (Fallon and Rozin, 1985; Cohn et al., 1987; Johnson and Engeln, 2021), indicating that IC in females may involve attempts to outcompete other females by fulfilling a preconceived conception of males’ desired body weight in their mates. This finding does not mean that only high BMI women will be prone to risky weight loss behavior. Indeed, eating disorder pathology can sometimes include being very low in BMI, and previous research has highlighted the intrasexually competitive nature of eating disorders. However, those with eating disorders typically view themselves as being fat and have poor body image, even though they are objectively thin (i.e., as in cases of anorexia nervosa). It is perhaps this common psychological perception of fatness that drives appearance-related risk-taking as a function of IC.

Results also showed that women who were higher in depression symptoms were more interested in using the risky diet pill. This finding aligns with other research from Canada showing that, whereas diet pill use is infrequent among the general population (0.5%), it is substantially higher among those with depression (~17%; Patten, 2001). Similar findings from the United States (National Longitudinal Study of Adolescent Health) found that adolescents who were higher in self-perceived weight and depression were more likely to take diet pills (Stephen et al., 2014). Research linking diet pill use and depression tends to view the association from the perspective of diet pills invoking a downstream risk of depression, such as through harmful effects on serotonin (e.g., McCann et al., 1997). However, our results highlight the novel empirical finding that depressed people may also be at risk for subsequently taking diet pills. In our exploratory model 1, this is further highlighted by the finding that depression (1) partially accounted for links between BMI and willingness to use a diet pill, and (2) that depression interacted with IC to predict willingness to use the risky diet pill, suggesting that diet pill use may be highest among those who are simultaneously depressed and intrasexually competitive. In exploratory model 2, we reversed the predictive roles of BMI and depression, and showed that higher BMI could also be considered as an outcome of depression, which might mediate links between depression and willingness to use a risky diet pill. In this manner, our data were limited by their cross-sectional design. Future research would benefit from conducting longitudinal work on young women by exploring BMI and depression at multiple timepoints and using path modeling to disentangle the relative directional influences of these variables, in relation with IC, toward intended diet pill use. Finally, beyond body weight, recent evidence has suggested that body shape, and not necessarily just body fat, predicts weight-based stigma. This indicates that there may be a difference depending on where the fat is distributed on women, which likely impacts perceived mate value (Krems and Neuberg, 2022). Considering body shape as an additional moderator to the link between IC and willingness to use a risky diet pill could provide a more robust understanding of how body shape might influence this relationship.

Further work should consider other relevant covariates not examined in this study to better inform the extent to which IC predicts women’s body image, dieting, and eating pathology. For example, other personality traits including perfectionism, poor interoceptive awareness, and obsessive-compulsiveness may be relevant to eating disorders (Lilenfeld et al., 2006, for review), but it is presently unclear whether some of these traits are also complicit in IC. Family dynamics may also be relevant to IC and eating disorder symptoms and pathologies. For instance, researchers have found a significant relationship between sibling rivalry in early childhood and IC (Buunk and Fisher, 2009). Further, Ahrén et al. (2013) found in a sample of 3,251 Swedish participants that females with a higher number of biological siblings displayed lower rates of eating disorders. Conversely, an increase in the number of half siblings positively correlated with the increased prevalence of eating disorders. The authors reasoned that diluted parental attention across more siblings might offer a protection effect for ‘parental demands’ that might normally promote eating disorders. An alternative evolutionary explanation is that perhaps a higher number of siblings is a proximate indicator of one’s mate value (e.g., Langenmayr and Schubert, 1990), which itself has been shown to relate to greater body satisfaction (Faer et al., 2005). These studies indicate that family dynamics can affect IC as well as the occurrence of eating disorders or body dissatisfaction in women and should be considered for in future research relating to diet pill interest. Mate value may be particularly relevant to these reported relationships given that previous research suggests that women who view themselves as less physically attractive relative to other women are more interested in engaging in appearance enhancement efforts, including taking risky diet pills (Arnocky, 2016).

One limitation of the present study is that we did not collect hormonal contraceptive use data. Hormonal contraceptives may also alter mating-relevant psychological processes, including IC (Alvergne and Lummaa, 2010; Welling et al., 2012). A two-part study examining dehumanization of rival females and IC revealed positive correlations between conception risk and dehumanization of same-sex rivals only for naturally ovulating females (Piccoli et al., 2013). Additionally, IC was higher among women with elevated conception risk who were not on any form of hormonal contraceptive (Piccoli et al., 2013). Lower levels of IC have also been established among pair-bonded women using hormonal contraceptives (Cobey et al., 2013). There is also limited evidence suggesting that the type of birth control plays a significant role in determining how IC is affected. Fergusson (2020) found that women using combined oral contraceptive pills reported higher levels of IC than females using progesterone-only methods. This suggests that certain hormonal contraceptives may have diverse effects on female IC.

Weight gain is a well-known side effect of many hormonal contraceptives, which contribute to an increase in BMI. As previously mentioned, higher BMI is significantly linked to increases in depressive symptoms and body dissatisfaction (Goldfield et al., 2010). Research has supported negative symptoms of hormonal contraceptives influencing body satisfaction. For example, Bird (2006) found some oral contraceptive side effects (e.g., mood symptoms and weight gain) significantly predicted body dissatisfaction and eating dysfunction while controlling for BMI and depression. Similarly, Bird and Oinonen (2011) found that women using oral contraceptives with a history of related mood and physical side effects had higher levels of body dissatisfaction and drive for thinness. Although research regarding hormonal contraceptives and body dissatisfaction is limited, initial research reveals some correlations which suggest that oral contraceptives could influence both IC and body image.

## Conclusion

Past research has identified links between women’s IC and body image distortion, potentially unhealthy weight control practices, including risky diet pill use. From this perspective, eating pathology and unhealthy weight control are a function of women’s adaptive competition with other women. Given men’s preference for attractive mates, women compete intrasexually within domains of physical attractiveness (see Davis and Arnocky, 2022, for review). Research on diet pill use has also been linked to real and self-perceived markers of body fat and depression, such that diet pill use tends to be higher among heavier and more depressed females. The present study extended upon this research by demonstrating that IC interacts with BMI in predicting willingness to use a risky diet pill, controlling for symptoms of depression. This finding suggests that links between IC and unhealthy weight control are not due to the potential confound of depression, but that it is contingent on BMI. Heavier women ostensibly have more to gain within the realm of IC by losing weight relative to thinner counterparts, and so may be more prone to engage in IC by employing risky weight loss tactics such as diet pill use.

## Data availability statement

The raw data supporting the conclusions of this article will be made available by the authors, without undue reservation.

## Ethics statement

The studies involving human participants were reviewed and approved by Nipissing University Research Ethics Board. Written informed consent from the participants’ legal guardian/next of kin was not required to participate in this study in accordance with the national legislation and the institutional requirements.

## Author contributions

SA funded the research, conceptualized the research question and design, conducted statistical analyses, and co-authored the manuscript. AD conducted analyses and co-authored the manuscript. HB and BD collected the data and co-authored the manuscript. All authors contributed to the article and approved the submitted version.

## Funding

Data collection costs supported by a grant from the Natural Sciences and Engineering Research Council of Canada (NSERC) Discovery Development Grant awarded to SA (file # DDG-2017-00013).

## Conflict of interest

The authors declare that the research was conducted in the absence of any commercial or financial relationships that could be construed as a potential conflict of interest.

## Publisher’s note

All claims expressed in this article are solely those of the authors and do not necessarily represent those of their affiliated organizations, or those of the publisher, the editors and the reviewers. Any product that may be evaluated in this article, or claim that may be made by its manufacturer, is not guaranteed or endorsed by the publisher.

## Footnotes

^1^Total proportion is greater than 100% because we allowed participants to select multiple ethnic/cultural backgrounds.
